# Supplementary material for: Selective Antibody-Free Sensing Membranes for Picogram Tetracycline Detection
Source: Biosensors (Basel). 2022 Dec 31;13(1):71. doi: 10.3390/bios13010071 (PMC9855611; doi:10.3390/bios13010071)
Supplement: Supplementary file 1 [file biosensors-13-00071-s001.zip › biosensors-2084853-supplementary.pdf]

biosensors

MDPI

Supplementary information

Selective Antibody-Free Sensing Membranes for Picogram Antibiotic Detection

Hamdi Ben Halima [1], Abdoullatif Baraket [1], Clara Vinas [2], Nadia Zine [1], Joan Bausells [3], Nicole Jaffrezic-Renault [1,*], Francesc Teixidor [2] and Abdelhamid Errachid [1]

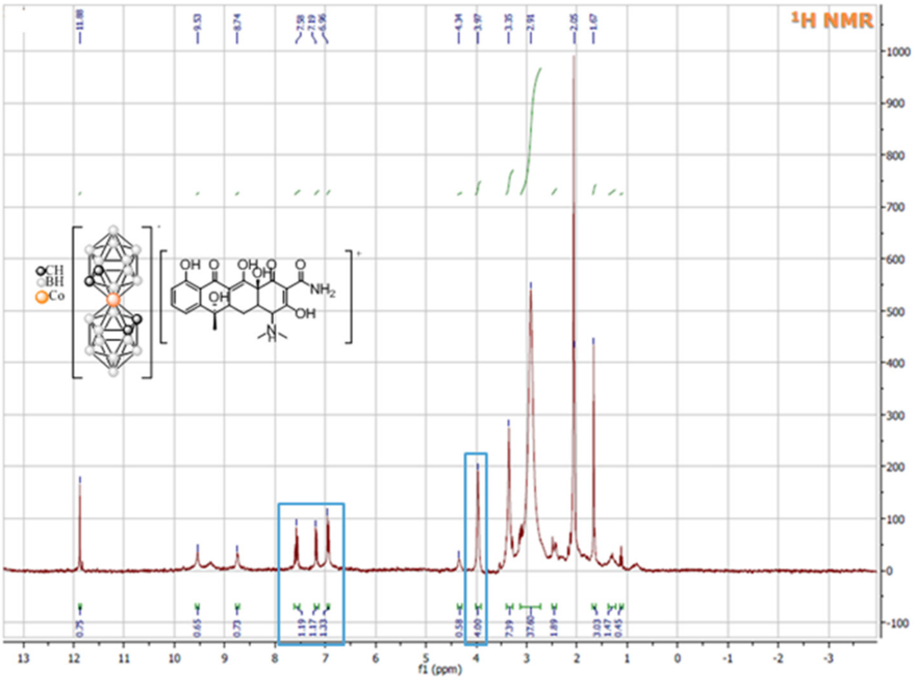

Figure S1. ^{1}H NMR spectrum of [*o*-COSAN]$^{-}$/tetracycline (deuterated acetone).

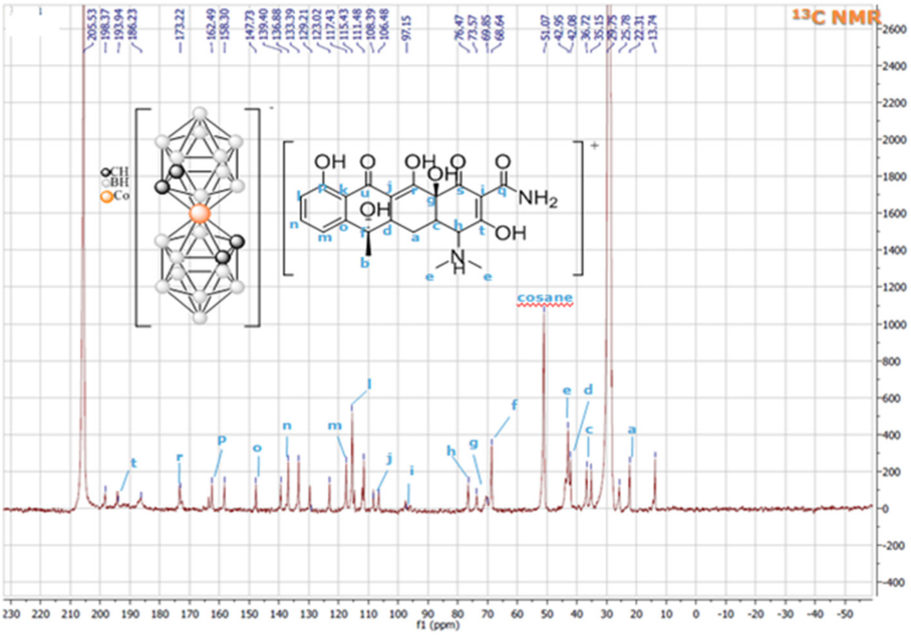

Figure S2. ^{13}C NMR spectrum of [*o*-COSAN]$^{-}$/tetracycline (deuterated acetone).

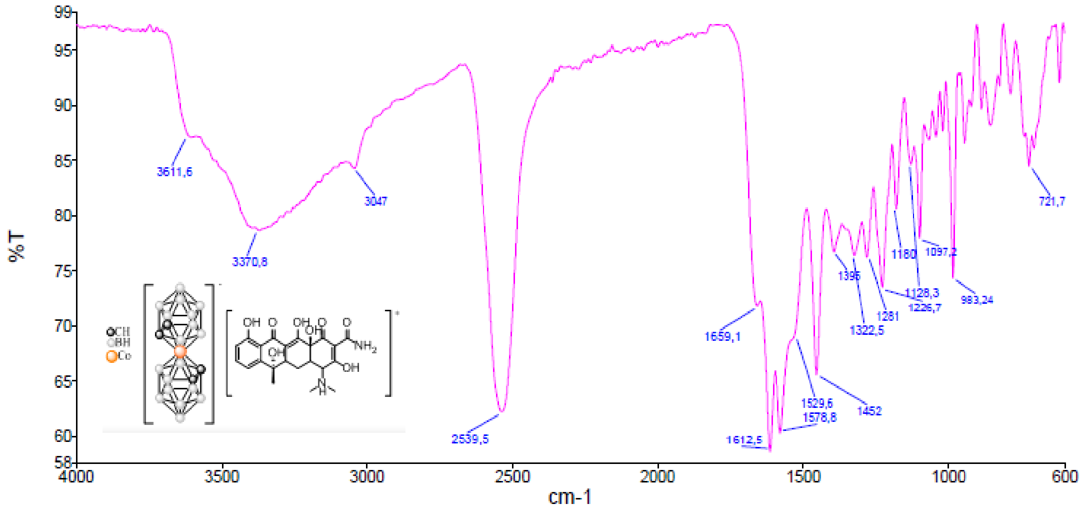

Figure S3. FTIR spectrum of [*o*-COSAN]⁻/tetracycline.

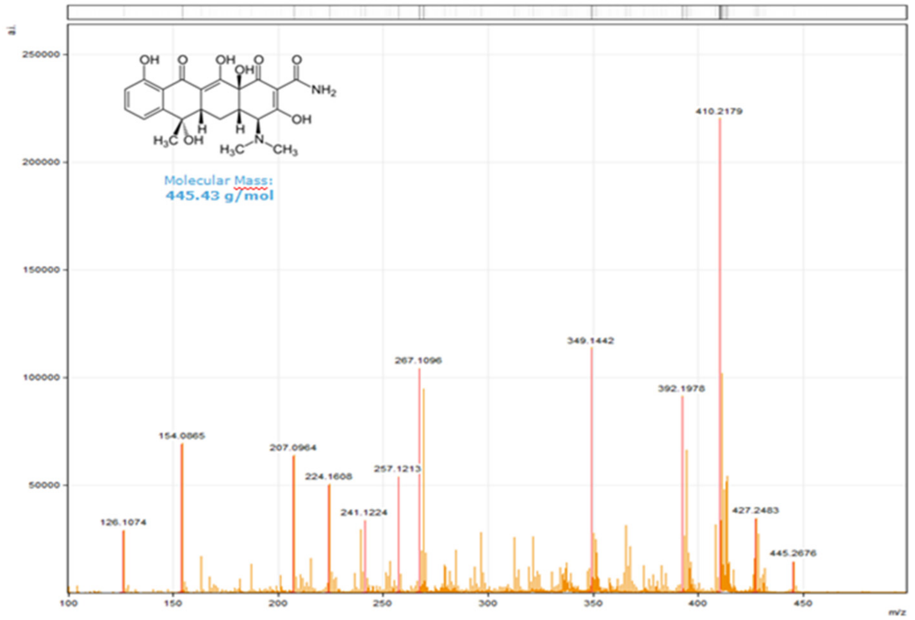

Figure S4. MALDI-TOF spectrum of [*o*-COSAN]⁻/tetracycline with matrix (positive part).

Table S1. Overview of elemental analysis in [*o*-COSAN]⁻/tetracycline.

		%C	%H	%N	%S
**Cosane/ Tetracycline**	Theory	40.52	6.23	3.64	0
	**Reality**	**40.00**	**6.00**	**3.50**	**0**

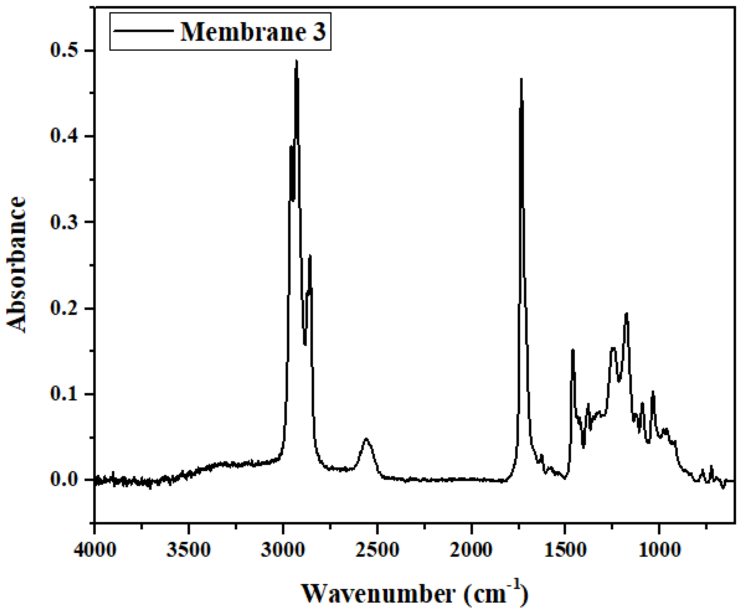

Figure S5. FTIR spectrum of membrane 3.

FTIR spectrum of membrane 3 displays the ν(N-H; O-H) at 3344-3400 cm^{-1}; ν(C-H) at 2859-2959 cm^{-1}; ν(B-H) at 2563 cm^{-1}; ν(C=O) at 1741 cm^{-1}; ν(C-H) at 1381-1464 cm^{-1}; ν($C=O_{amide}$; C=C; $C=C_{aromatique}$; N-H) at 1451-1498 cm^{-1}; ν(C-H) at 1325-1451 cm^{-1}; ν(C-O; C-C; C-N) at 1019-1325 cm^{-1}.

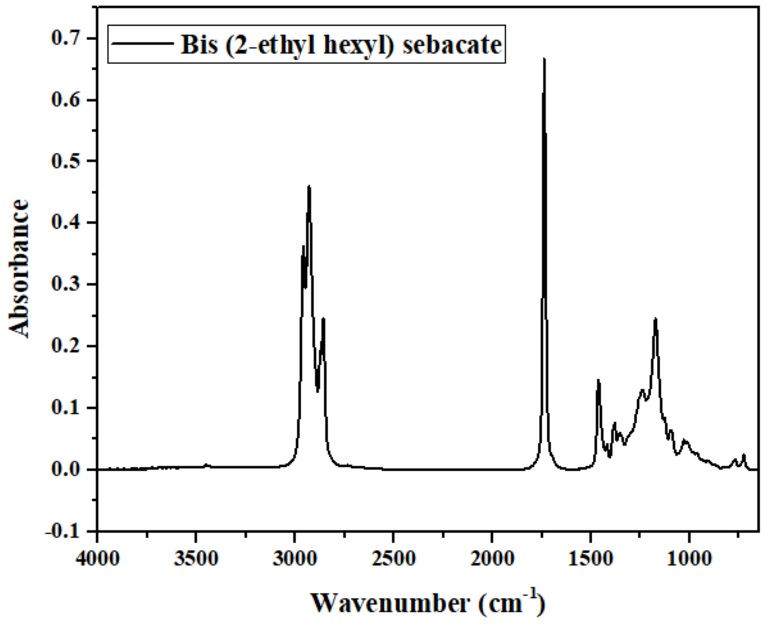

Figure S6. FTIR spectrum of bis (2-ethyl hexyl) sebacate.

FTIR spectrum of bis (2-ethyl hexyl) sebacate displays the ν(C-H) at 2859-2959 cm^{-1}; ν(C=O) at 1739 cm^{-1}; ν(C-H) at 1381-1464 cm^{-1}; ν(C-C) at 1030-1241 cm^{-1}.

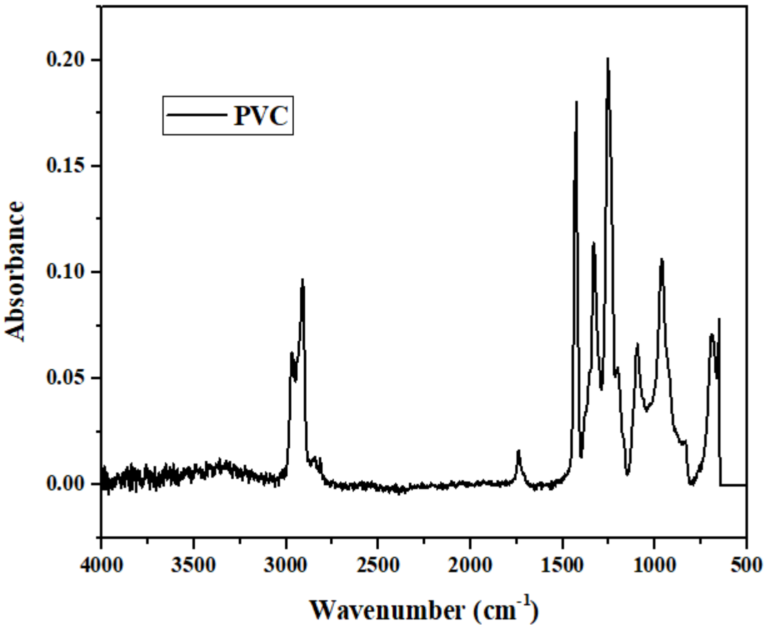

Figure S7. FTIR spectrum of PVC.

FTIR spectrum of PVC displays the ν(C-H) at 2911-2970 cm^{-1}; ν(C-H) at 1332-1427 cm^{-1}; ν(C-C) at 1094-1255 cm^{-1}; ν(C-Cl) at 689 cm^{-1}.

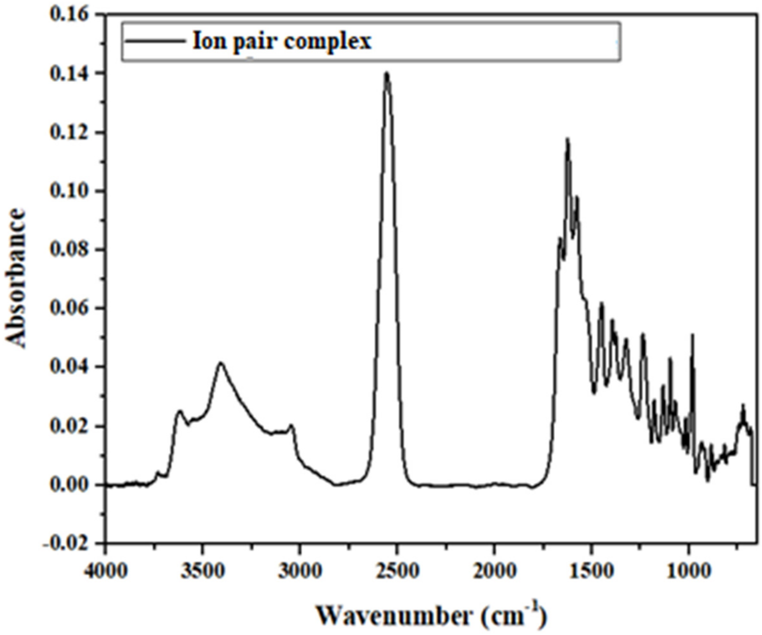

Figure S8. FTIR spectrum of [*o*-COSAN]$^-$/tetracycline ion pair complex.

FTIR spectrum of ionophore of cobaltabisdiscarbollide displays a strong and characteristic ν(B-H) frequency in the infrared range 2556 cm^{-1} in which no other frequencies of organic compounds appear. The FTIR spectrum displays the ν (O-H) stretching vibration at 3617 cm^{-1}; ν(N-H; O-H) at 3411-3617 cm^{-1}; ν(C-H) at 3050 cm^{-1}; ν(B-H) at 2556 cm^{-1}; ν(C=O_{amide}; C=C; C=$C_{aromatique}$; N-H) at 1451-1663cm^{-1}; ν(C-H) at 1325-1451 cm^{-1}; ν(C-O; C-C; C-N) at 1019-1325 cm^{-1}; ν(C-H; C=C) at 723-983 cm^{-1}.

Table S2. Fitting parameters obtained from Randles equivalent circuit model [$R_s + Q_2/ (Rct + Z_W)$] (membrane 3 detection of tetracycline).

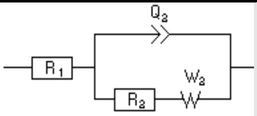	**R1 (kΩ)**	**Q2 (nF.s$^{(a-1)}$)**	**R2 (kΩ)**	**s2 (kΩ.s$^{-1/2}$)**	X^2
PBS	$3687 \times 10^{-3} \pm 0.6877 \times 10^{-3}$	$0.1119 \times 10^{-9} \pm 4.294 \times 10^{-15}$	$0.936253 \times 10^{3} \pm 1.856 \times 10^{-3}$	$1.588 \times 10^{3} \pm 14.86 \times 10^{-3}$	4.125×10^{-3}
1 pg/L	$11706 \times 10^{-3} \pm 0.2448 \times 10^{-3}$	$0.1357 \times 10^{-9} \pm 1.475 \times 10^{-15}$	$2.943 \times 10^{3} \pm 1.901 \times 10^{-3}$	$2.753 \times 10^{3} \pm 14.52 \times 10^{-3}$	8.571×10^{-3}
50 pg/L	$10822 \times 10^{-3} \pm 0.3578 \times 10^{-3}$	$0.1374 \times 10^{-9} \pm 0.7108 \times 10^{-15}$	$5.706 \times 10^{3} \pm 1.88 \times 10^{-3}$	$5.041 \times 10^{3} \pm 11.51 \times 10^{-3}$	5.612×10^{-3}
0.1 ng/L	$16655 \times 10^{-3} \pm 0.4266 \times 10^{-3}$	$0.1438 \times 10^{-9} \pm 0.5499 \times 10^{-15}$	$8.116 \times 10^{3} \pm 1.753 \times 10^{-3}$	$7.496 \times 10^{3} \pm 14.19 \times 10^{-3}$	6.395×10^{-3}
0.5 ng/L	$8611 \times 10^{-3} \pm 0.2921 \times 10^{-3}$	$0.1472 \times 10^{-9} \pm 0.55541 \times 0^{-15}$	$9.762 \times 10^{3} \pm 2.352 \times 10^{-3}$	$9.148 \times 10^{3} \pm 16.66 \times 10^{-3}$	3.488×10^{-3}
1 ng/L	$23889 \times 10^{-3} \pm 0.3422 \times 10^{-3}$	$0.148 \times 10^{-9} \pm 0.6116 \times 10^{-15}$	$11.07 \times 10^{3} \pm 3.405 \times 10^{-3}$	$11.57 \times 10^{3} \pm 34.21 \times 10^{-3}$	2.745×10^{-3}
1.5 ng/L	$5835 \times 10^{-3} \pm 0.4588 \times 10^{-3}$	$0.143910^{-9} \pm 0.5297 \times 10^{-15}$	$12.33 \times 10^{3} \pm 4.284 \times 10^{-3}$	$13.61 \times 10^{3} \pm 35.79 \times 10^{-3}$	2.454×10^{-3}
2ng/L	$15 \pm 0.5623 \times 10^{-3}$	$0.1432 \times 10^{-9} \pm 0.3862 \times 10^{-15}$	$13.37 \times 10^{3} \pm 3.056 \times 10^{-3}$	$12.64 \times 10^{3} \pm 25.41 \times 10^{-3}$	3.085×10^{-3}
3ng/L	$15636 \times 10^{-3} \pm 0.5435 \times 10^{-3}$	$0.145 \times 10^{-9} \pm 0.328 \times 10^{-15}$	$14.36 \times 10^{3} \pm 1.939 \times 10^{-3}$	$13.45 \times 10^{3} \pm 19.36 \times 10^{-3}$	3.955×10^{-3}
4ng/L	$15317 \times 10^{-3} \pm 0.5591 \times 10^{-3}$	$0.1408 \times 10^{-9} \pm 0.2865 \times 10^{-15}$	$15.86 \times 10^{3} \pm 2.505 \times 10^{-3}$	$15.39 \times 10^{3} \pm 20.17 \times 10^{-3}$	5.033×10^{-3}
5ng/L	$-28246 \times 10^{-3} \pm 0.631 \times 10^{-3}$	$0.1503 \times 10^{-9} \pm 0.3127 \times 10^{-15}$	$17.23 \times 10^{3} \pm 4.832 \times 10^{-3}$	$16.74 \times 10^{3} \pm 29.11 \times 10^{-3}$	2.156×10^{-3}

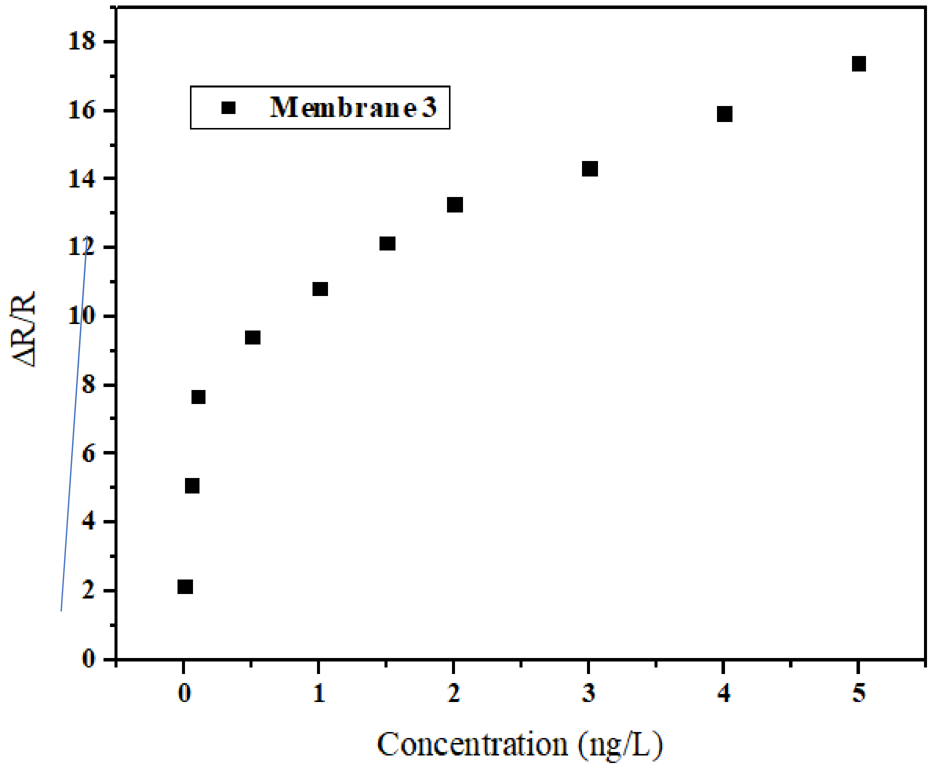

Figure S9. Calibration curve of the tetracycline sensor with membrane 3.
